# Clinical dilemma of mood stabilizer augmentation in treatment‐resistant schizophrenia with residual phase: A case report of valproate‐associated hypoglycemia and cytopenia, and subsequent lithium response

**DOI:** 10.1002/pcn5.70334

**Published:** 2026-04-17

**Authors:** Yoshiki Kasagi, Naomichi Okamoto, Reiji Yoshimura

**Affiliations:** ^1^ Department of Psychiatry University of Occupational and Environmental Health Kitakyushu Fukuoka Japan

**Keywords:** lithium, mood stabilizer, residual phase, schizophrenia, valproic acid

## Abstract

**Background:**

Mood stabilizer augmentation is frequently used in treatment‐resistant schizophrenia despite limited guideline recommendations. Evidence for its efficacy and safety in the residual phase, however, remains insufficient.

**Case Presentation:**

A 49‐year‐old Japanese woman with treatment‐resistant residual schizophrenia exhibited persistent agitation and aggression despite antipsychotic therapy. Valproate augmentation (800 mg/day) improved irritability and psychomotor agitation but was complicated by hypoglycemia, suggestive of relative carnitine deficiency, and subsequent pancytopenia. Both adverse events resolved after discontinuation. Behavioral symptoms markedly worsened following withdrawal. Clozapine and electroconvulsive therapy were declined. Lithium augmentation (600 mg/day; serum level 0.8 mEq/L) was initiated, resulting in improvement of dangerous behaviors and agitation. The Brief Psychiatric Rating Scale total score improved from 80 to 64, particularly in the hostility and excitement items. No readmission to the psychiatric ward occurred during a 3‐month follow‐up.

**Conclusion:**

This case highlights the clinical dilemma of mood stabilizer augmentation in residual‐phase schizophrenia. While valproate demonstrated behavioral efficacy, serious adverse events necessitated its discontinuation. Lithium may represent a potential alternative for managing aggression and agitation, but further research is warranted.

## BACKGROUND

Antipsychotics remain the cornerstone of schizophrenia treatment. However, approximately 30% of patients continue to experience persistent psychotic symptoms, including hallucinations, delusions, agitation, and irritability, despite adequate antipsychotic therapy. In treatment‐resistant cases where clozapine or electroconvulsive therapy is not indicated, feasible, or accepted, adjunctive mood stabilizers are sometimes added to antipsychotic regimens to alleviate residual symptoms.^1^, ^2^

Current clinical practice guidelines do not actively recommend the use of mood stabilizers in patients with schizophrenia, primarily because of the lack of high‐quality evidence supporting their efficacy.^3^ Nevertheless, real‐world data indicate that antipsychotic–mood stabilizer combination therapy is relatively common, with reported rates ranging from 14.1% to 40.7%.^1^ One possible explanation for the inconsistent therapeutic effects of mood stabilizer augmentation is the heterogeneity of schizophrenia. Schizophrenia is not a single, uniform disease entity but rather a spectrum of disorders encompassing diverse symptom profiles and illness phases—from acute to chronic and residual—and, in some cases, overlapping with schizoaffective disorders through affective symptom dimensions.^4^

Compared with the evidence supporting mood stabilizer use in schizoaffective disorders, longitudinal reports describing both therapeutic outcomes and adverse events in patients with treatment‐resistant residual schizophrenia remain limited. In this report, we describe a patient with treatment‐resistant residual schizophrenia in whom valproate augmentation was associated with an improvement in agitation but also led to severe adverse events, including hypoglycemia and pancytopenia, followed by behavioral improvement after subsequent lithium augmentation.

## CASE PRESENTATION

A 49‐year‐old Japanese woman was referred to our hospital for management of poorly controlled psychiatric symptoms. There was no family history of any psychiatric disorders, including bipolar disorder. Her medical history was notable for hypothyroidism, treated with levothyroxine sodium 25 μg daily. At the time of referral, she was receiving zotepine 300 mg/day.

She first became aware of contamination concerns at approximately 12 years of age. By 15 years, she exhibited compulsive handwashing, psychomotor slowing, and unusual behaviors, including walking sideways along the road. Around 17 years, she developed soliloquy, inappropriate smiling, violent behavior, and neglect of hygiene. She subsequently refused psychiatric care and became socially withdrawn, confining herself to her room.

At 35 years of age, she was brought to our hospital by her family. She exhibited persistent psychotic symptoms, including auditory hallucinations, disorganized behavior, aggression, and marked social withdrawal, meeting the Diagnostic and Statistical Manual of Mental Disorders, Fifth Edition criteria for schizophrenia. The criteria for schizoaffective disorder were not met, and no lifetime history of manic/hypomanic episodes was noted based on longitudinal history and collateral information. She was subsequently treated with antipsychotics, including quetiapine up to 800 mg/day and olanzapine up to 20 mg/day; however, bizarre behavior, poor hygiene, irritability, and aggression persisted. Clozapine and electroconvulsive therapy were proposed but declined by her family. Valproate 800 mg/day was added to olanzapine, resulting in improved irritability and agitation, although hallucinations and delusions persisted. She continued to live at home for some time, but her functional status gradually declined, and she was admitted to a residential care facility at 42 years of age.

At 47 years of age, she developed hypoglycemia. A comprehensive evaluation was conducted to identify potential causes, including hepatic dysfunction, sepsis, adrenal insufficiency, thyroid dysfunction, and insulin autoimmune syndrome; however, all were excluded. Serum carnitine analysis revealed free carnitine 22.5 µmol/L, acylcarnitine 3.0 µmol/L, and an acylcarnitine/free carnitine ratio of 0.13, all at the lower limit of the normal range. Although the diagnostic criteria for carnitine deficiency—defined as (i) free carnitine < 20 µmol/L, (ii) acylcarnitine ≥ 20 µmol/L, or (iii) acylcarnitine/free carnitine ratio > 0.4—were not met, the clinical course and laboratory findings suggested a probable prodromal or relative carnitine deficiency state. Given the long‐term administration of valproate, it was suspected to be the causative agent. Valproate was discontinued, and l‐carnitine supplementation was initiated. Subsequently, serum‐free carnitine levels increased to the normal mean range, and hypoglycemic symptoms resolved.

However, following valproate discontinuation, she developed severe behavioral disturbances, including kicking walls and biting staff members, accompanied by marked psychomotor agitation. Valproate was re‐administered, resulting in temporary improvement of psychiatric symptoms; however, pancytopenia subsequently developed, with a white blood cell count of 2870/µL, hemoglobin of 8.8 g/dL, and platelet count of 2.4 × 10^3^/µL. After valproate discontinuation, hematological abnormalities improved. Nevertheless, psychotic symptoms markedly worsened, including increased soliloquy, inappropriate laughter, shouting, and aggression toward other residents, rendering continued care at the facility difficult. She was subsequently referred to and admitted to our hospital.

Upon admission, she exhibited blunted and disorganized speech. She was able to respond to the closed‐ended questions; however, her answers to open‐ended questions were limited and often poorly understood. Upon entering the ward, she displayed marked psychomotor agitation with dangerous behaviors, including loud shouting, attempting to leave the ward despite staff restraint, trying to bite staff members, and scratching herself. After being escorted to her room, she continued to exhibit bizarre behaviors, such as standing motionless with her head down, licking her fingers individually, rubbing her clothing while facing staff, and performing repetitive brushing or wiping gestures directed toward empty space. Her total Brief Psychiatric Rating Scale (BPRS) total score was 80.

Laboratory tests revealed hyperprolactinemia (184 ng/mL), with no other significant abnormalities in endocrine parameters, electrolytes, liver function tests, or vitamin levels. Electroencephalography was unremarkable, and brain magnetic resonance imaging showed mild medial temporal lobe atrophy without other notable findings.

Zotepine dose was increased from 300 to 450 mg/day to address psychomotor agitation; however, symptoms such as loud shouting persisted. Clozapine and electroconvulsive therapy were again proposed to the family, but they declined. Lithium carbonate was initiated at 400 mg/day and titrated to 600 mg/day. When serum lithium levels reached 0.8 mEq/L, the previously observed dangerous behaviors and shouting resolved, and bizarre behaviors decreased. At discharge, the BPRS score had improved to 64, with notable reductions in Hostility and Excitement (Table 1). At the 3‐month outpatient follow‐up, hallucinations and delusions persisted; however, agitation and violent behavior were not observed.

**Table 1:** Brief Psychiatric Rating Scale (BPRS) scores.

Item	Before lithium treatment	After lithium treatment
Somatic concern	3	2
Anxiety	5	4
Emotional withdrawal	5	4
Conceptual disorganization	6	6
Guilt feelings	2	2
Tension	5	4
Mannerisms and posturing	6	5
Grandiosity	3	3
Depressive mood	2	2
Hostility	6	2
Suspiciousness	5	2
Hallucinatory behavior	6	5
Motor retardation	3	3
Uncooperativeness	4	4
Unusual thought content	5	4
Blunted affect	4	4
Excitement	6	4
Disorientation	4	4
Total	80	64

*Note*: Lithium augmentation was associated with notable improvements, particularly in Hostility and Excitement items of the BPRS.

## DISCUSSION AND CONCLUSION

This case highlights the clinical dilemma of mood stabilizer augmentation in a patient with treatment‐resistant schizophrenia during the residual phase, characterized by persistent symptoms.

In patients with schizophrenia, adequate symptom control is often not achieved with antipsychotic monotherapy. In such cases, mood stabilizers are frequently added in real‐world clinical practice, despite the lack of strong recommendations in current clinical guidelines.^1^, ^3^ The agents most commonly used for this purpose include valproate, lithium, lamotrigine, and carbamazepine.^2^, ^5^, ^6^, ^7^

Valproic acid, a GABAergic agent, is thought to facilitate dopaminergic downregulation^8^ and may, therefore, be particularly effective in targeting specific symptoms, such as agitation and aggression.^2^ In this case, reproducible exacerbation of marked psychomotor agitation and aggression occurred after valproate discontinuation, followed by symptomatic improvement upon reintroduction, consistent with a dechallenge–rechallenge pattern and supporting its clinical efficacy for behavioral dysregulation. However, compared with control groups, valproate has been associated with a significantly higher incidence of adverse events.^2^ Hematological abnormalities, including thrombocytopenia, leukopenia, and anemia, have been reported as rare but clinically significant adverse effects.^9^, ^10^, ^11^ Mechanistically, valproate has been proposed to induce DNA damage through chronic inhibition of histone deacetylases and to promote oxidative metabolism via free radical intermediates, potentially leading to reversible myelodysplastic changes.^10^, ^12^, ^13^ In addition, valproate is known to reduce systemic carnitine stores and impair mitochondrial β‐oxidation, thereby increasing the risk of hypoglycemia.^14^, ^15^ Because β‐oxidation is essential for energy production during fasting states, carnitine deficiency–related impairment of β‐oxidation may result in insufficient ATP generation and subsequent hypoglycemia. Based on three criteria of the World Health Organization–Uppsala Monitoring Center Causality Assessment System—namely, a reasonable temporal relationship between drug exposure and the event, the absence of alternative explanations, and clinically plausible improvement following drug withdrawal—we assessed valproate as the probable causative agent in this case.^16^

The fundamental mechanisms underlying the mood‐stabilizing effects of lithium remain incompletely understood. However, lithium is thought to attenuate enhanced dopaminergic activity through its action on the β‐arrestin complex, while also reducing synaptic glutamate levels and promoting inhibitory neurotransmission.^17^ In addition, lithium influences intracellular signaling pathways via inhibition of glycogen synthase kinase‐3 and modulation of neurotrophic factors.^18^ These multifaceted effects may contribute to the stabilization of agitation and behavioral dysregulation. A Cochrane review of 13 studies reported that participants receiving lithium augmentation in addition to antipsychotics showed significantly better clinical response than those receiving antipsychotic monotherapy (10 randomized controlled trials [RCTs], *N* = 396; risk ratio 1.81, 95% confidence interval [CI] 1.10–2.97).^5^ In one of the included RCTs, a favorable statistical trend for lithium was observed, specifically in Positive and Negative Syndrome Scale aggression scores.^5^ However, when participants with schizoaffective disorder, non–double‐blind trials, or studies with high attrition rates were excluded, the superiority of lithium augmentation was no longer statistically significant.^5^ In a large nationwide cohort study from Finland including all patients hospitalized for schizophrenia between 1972 and 2014 (*N* = 61,889), lithium augmentation was associated with a lower risk of psychiatric rehospitalization compared with antipsychotic monotherapy (hazard ratio 0.83, 95% CI 0.81–0.85).^1^ However, this study was unable to assess symptom severity or longitudinal symptom fluctuations and did not include patients treated exclusively in outpatient settings, which limits interpretation. In the present case, improvement in the hostility and excitement items of the BPRS was consistent with prior findings, suggesting a potential benefit of lithium for aggression and behavioral dysregulation. Nevertheless, the influence of confounding factors—such as zotepine dose escalation, structured inpatient environment, and natural course over time—cannot be excluded.

This study is limited by its single‐case design, and a direct causal relationship between mood stabilizer augmentation and clinical improvement cannot be definitively established. Nonetheless, this case emphasizes that mood stabilizer augmentation in patients with chronic treatment‐resistant schizophrenia should be approached cautiously and tailored to specific symptom profiles. Furthermore, when the therapeutic benefit is unclear or significant adverse events occur, prompt dose reduction or discontinuation should be considered. Importantly, before diagnosing treatment‐resistant schizophrenia and considering mood stabilizer augmentation, potential sources of pseudo‐resistance should be carefully excluded, including poor medication adherence, substance use, and psychiatric or medical comorbidities. Given the frequent real‐world use of mood stabilizer augmentation in treatment‐resistant schizophrenia, further high‐quality studies evaluating their efficacy and safety are warranted.

## AUTHOR CONTRIBUTIONS

Yoshiki Kasagi and Naomichi Okamoto contributed to study conception and design, data acquisition, and data analysis and interpretation. Reiji Yoshimura supervised the study and critically revised the manuscript. All authors approved the final manuscript.

## CONFLICT OF INTEREST STATEMENT

The authors declare no conflicts of interest.

## ETHICS APPROVAL STATEMENT

All procedures described in this case report were conducted in accordance with ethical guidelines. As this is a single‐case report, the Ethics Committee of the University of Occupational and Environmental Health waived the need for formal review.

## PATIENT CONSENT STATEMENT

Written informed consent was obtained from the patient for participation in this study.

## CLINICAL TRIAL REGISTRATION

Not applicable.

## Data Availability

The data that support the findings of this study are available from the corresponding author upon reasonable request.
